# Effect of Oral Intake of Carrot Juice on Cyclooxygenases and Cytokines in Healthy Human Blood Stimulated by Lipopolysaccharide

**DOI:** 10.3390/nu15030632

**Published:** 2023-01-26

**Authors:** Ulrik Deding, Bettina Hjelm Clausen, Issam Al-Najami, Gunnar Baatrup, Boye Lagerbon Jensen, Morten Kobaek-Larsen

**Affiliations:** 1 Department of Clinical Research, University of Southern Denmark, 5230 Odense, Denmark; 2 Department of Surgery, Odense University Hospital, 5000 Odense, Denmark; 3 Department of Neurobiology Research, Institute of Molecular Medicine, University of Southern Denmark, 5000 Odense, Denmark; 4 Cardiovascular and Renal Research, University of Southern Denmark, 5000 Odense, Denmark

**Keywords:** falcarinol, polyacetylenes, inflammation, *ex vivo*, anti-neoplastic

## Abstract

*In vitro* studies and animal studies have shown that chemical compounds contained in carrots, such as falcarinol and falcarindiol, can prevent inflammation. The present study was designed to test whether the oral intake of carrot juice containing falcarinol and falcarindiol affects the activity of cyclooxygenase (COX) enzymes and the secretion of inflammatory cytokines in human blood. Carrot juice (500 mL) was administered orally to healthy volunteers, and blood samples were drawn before and 1 h after juice intake at the time point when peak concentrations of falcarinol and falcariondiol have been shown in the blood. The blood samples were divided, and one sample was allowed to coagulate for 1 h at room temperature before analyzing the synthesis of thromboxane B2 (TBX2) by the COX1 enzyme using an enzyme linked immunosorbent assay (ELISA). The other blood samples were stimulated *ex vivo* with lipopolysaccharide and incubated at 37 °C for 24 h. The ELISA and cytokine multiplex analysis assessed the levels of COX-2-induced prostaglandin E2 (PGE2) and inflammatory markers interleukin (IL) 1α, IL1β, IL6, IL16, and tumor necrosis factor α (TNFα). Inflammatory cytokines such as IL1α and IL16 were significantly reduced in the LPS stimulated blood samples with higher concentrations of falcarinol and falcariondiol compared to the control samples taken before the intake of carrot juice. The levels of TBX2, PGE2, IL1β, IL6, and TNFα were not affected by the carrot juice intake blood samples not stimulated with LPS. In conclusion, carrot juice rich in the polyacetylens falcarinol and falcarindiol affects blood leukocytes, priming them to better cope with inflammatory conditions, evident by the reduced secretion of the proinflammatory cytokines IL1α and IL16.

## 1. Introduction

Polyacetylenes falcarinol (FaOH) and falcarindiol (FaDOH) are plant-derived acetylenic phyto-oxylipins available in the diet through plants from the Apiaceae family, which include carrot (*Daucus carota* L.), parsnip (*Pastinaca satica*), celery (*Apium graveolens* L.), fennel (*Foeniculum vulgare*), and some herbs, such as parsley root (*Petroselinum crispum*) and lovage root (*Levisticum officinale*). Falcarinol is the primary polyacetylene metabolite from which other forms, such as falcarindiol and falcarindiol-3-acetate, are derived. Among these food sources, carrot is the most widely consumed vegetable, contributing most to the dietary intake of FaOH [1,2,3]. However, the FaOH content in carrots varies, as levels are affected by growing conditions and cultivar [4]. Carrot FaOH content has been reported to be between 0.6 mg and 6.7 mg/100 g fresh weight [5,6,7,8] and typically varies between 100 and 600 mg/kg dry weight (DW) [9]. We have previously demonstrated that the intake of 30 g freeze-dried carrot diluted in 500 mL tap water resulted in a peak concentration of 4.0 ng FaOH per ml serum 1 h after intake [5]. The intake of 300 g of raw carrots may reach concentrations in the blood circulation, which have been demonstrated to affect the growth of human cells *in vitro* [1,6,7,8,10,11]. 

Anti-inflammatory drugs are one of the most commonly used categories of drugs for human use. The inhibition of inflammation plays a major role in pain control and in acute and chronic inflammatory processes observed during trauma, rheumatic and autoimmune conditions. Non-steroidal drugs (NSAIDs), such as aspirin, have been demonstrated to significantly affect the primary and secondary prevention of cardiovascular disease and cancer. These drugs are inhibitors of the cyclooxygenase (COX) enzymes. Two COX enzymes, with similar kinetics and substrate specificity, catalyze the formation of prostaglandin E2 (PGE2) and thromboxane B2 (TXB2) from arachidonic acid. Prostaglandin G2 and H2 is short-lived and rapidly converted to biologically active prostanoids by the cell-specific enzyme COX-1, which also catalyzes the formation of TXB2 in thrombocytes. The COX-1 enzyme is a housekeeping enzyme, constitutively expressed throughout the body and of particular importance for gastrointestinal mucosa protection [12]. COX-2 catalyzes the formation of PGE2 in many cells. COX-2 levels are low but are rapidly induced in early response to growth factors, cytokines, and tumor promoters following inflammation, abnormal proliferation, angiogenesis, cell invasion, or cancer metastasis. An association between colorectal cancer and COX-2 overexpression has been suggested [13,14]. 

In rodent studies, oral administration of FaOH and FaDOH has been shown to down-regulate Nuclear Factor κB, COX-2, IL-6, tumor necrosis factor α (TNFα), but not COX-1 and IL-1β gene expression in the neoplastic colonic tissue [15]. In mice, one week of oral FaOH intake before lipopolysaccharide (LPS) injection resulted in a T-helper cell (Th2/Th9) response, ensuing increased plasma levels of anti-inflammatory cytokine [16,17]. 

Two key players of inflammation are interleukin (IL) 1α (IL-1α), produced by macrophages and neutrophils, and IL-16, primarily produced by lymphocytes. In general, IL-1α is a damage-associated molecular pattern-induced cytokine, which evokes many inflammatory reactions via the IL-1 receptor type 1 (IL-1R1) [18]. IL-16 is a multifunctional cytokine that plays a fundamental role in inflammatory diseases and the development and progression of various cancers. 

This study aimed to translate the findings obtained from rodent studies into a human setting. We tested whether the oral intake of carrot juice influences the immunomodulatory effect of blood-circulating immune cells to better cope with an inflammatory condition. Carrot juice contains several nutrients and non-nutrients that affect inflammation; however, in this study, we focus on the effect of polyacetylens. We analyzed key proteins (PGE2, IL-1α, IL-1β, IL-6, IL-16, and TNFα) after LPS stimulation using a human *ex vivo* whole blood assay. Furthermore, we analyzed whether the thrombocyte COX-1 activity is affected. 

## 2. Materials and Methods

### 2.1. Study Subjects

**Figure 1:**
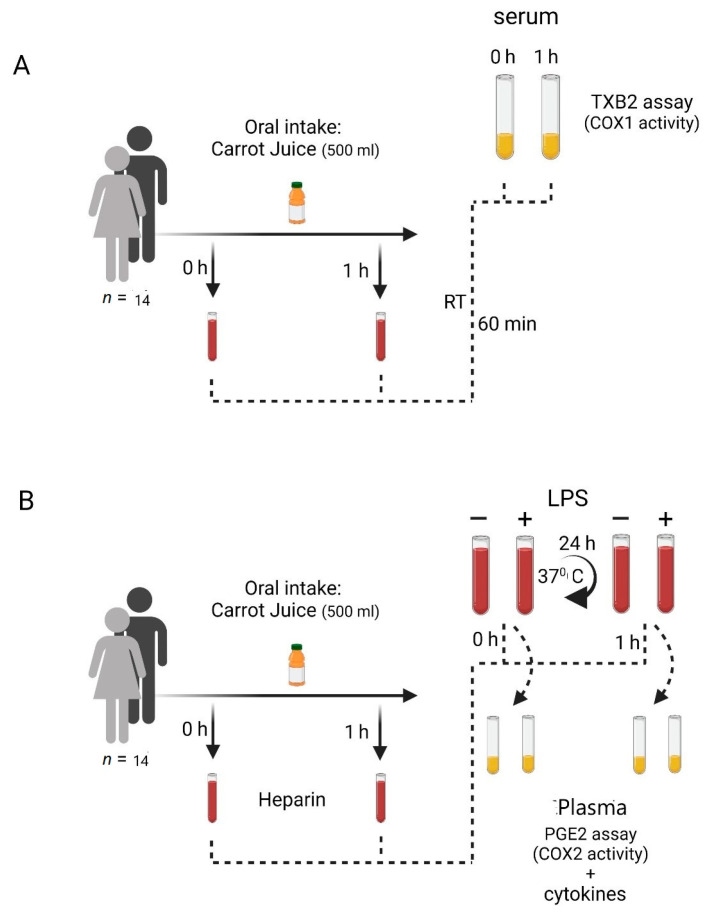
Study flow (**A**) Preparation of serum samples for analysis of thrombocyte COX-1 activity. (**B**) Preparation of plasma samples for analysis of inflammatory response (PGE2, IL-1α, IL-1β, IL-6, IL-16 and TNFα) to LPS. Cyclooxygenase (COX), Tromboxane (TX), Prostaglandin (PL), Interleukin (IL), Tumor Necrosis Factor (TNF).

Fourteen healthy volunteers (9 females and 5 males, aged 20–55 years) were included in the study. The subjects’ food intake was not restricted before the study; however, they had not ingested NSAIDs, other anti-inflammatory agents, or vegetables containing FaOH and FaDOH, including carrots, for 48 h preceding the study. The participants received oral and written information about the study and signed a consent agreement before inclusion. Figure 1 illustrates the experimental setup of the study. This study was approved by the Regional Health Research Ethics Committee (S-20210071).

### 2.2. Carrot Juice

The carrots (cv. Night Bird F1 hybrid) were grown organically at Danroots A/S (Bjerringbro, Denmark). The tops and bottoms were removed from fresh, washed carrots, which were subsequently shredded, freeze-dried, and prepared into powder (European Freeze-Dry, Kirke Hyllinge, Denmark). The powder was packed in sealed aluminum foil pouches and stored at −30 °C until further use. Next, the carrot juice was prepared in a plastic shaker on the day of oral intake. The juice was prepared by reconstitution 30 g of freeze-dried carrot powder (cv. Night Bird F1 hybrid) in 500 mL of tap water. The juice was administrated orally within 10 min. As previously shown, the slurry of 30 g carrot powder gave rise to a maximum concentration of FaOH in the blood serum 1 h after intake [5].

### 2.3. Blood Sampling 

For each individual, 2 times 4 milliliter aliquots of peripheral venous blood samples were transferred into glass tubes (serum samples) and 3 times 4 mm aliquots of peripheral venous blood samples were transferred into glass tubes containing 10 IU of sodium heparin (plasma samples) before the oral intake of the carrot juice. This sampling was repeated 1 h after the oral intake of the carrot juice.

### 2.4. Blood Handling

The *ex vivo* assays used for measuring COX-1 and COX-2 activity in this study were previously described by Patrignani and colleagues [19].

#### 2.4.1. COX-1 Activity in Human Blood

Platelet COX-1 acetylation mirrors serum TXB2 formation [20]; therefore, TXB2 can be used as a measure of platelet COX-1 enzyme activity during the blood clotting process. The human whole blood samples were allowed to clot at room temperature for 60 min. Next, the serum was separated by centrifugation (10 min at 2000 rpm) and kept at −30 °C until being assayed for TXB2 content. The whole blood TXB2 production was measured as a reflection of COX-1 activity (Figure 1A). Aliquots of 4 mL containing 1 mg/mL acetylsalicylic acid (ASA) (PHR1003, Aspirin (Acetyl Salicylic Acid, MERCK) were used as a negative control for COX-1 activity [19,21,22].

#### 2.4.2. COX-2 Induction in Human Blood

Four milliliter aliquots of human whole blood containing 10 IU of sodium heparin were incubated in either the absence or presence of LPS (10 microgram/mL) (E. coli O111:B4, MERCK) for 0 to 24 h, at 37 °C. The plasma was separated by centrifugation (10 min at 2000 rpm) and kept at −30 °C until being assayed for PGE2 (Figure 1B). Aliquots containing 10 μg Dexamethasone (D1756, MERCK) per ml were used as a negative control for COX-2 activity [21,22].

#### 2.4.3. Cytokine Induction in Human Blood

Four milliliter aliquots of whole blood samples containing 10 IU of sodium heparin were incubated in both the absence and presence of LPS (10 μg/mL) (E. coli O111:B4, MERCK) for 0 to 24 h at 37 °C. The plasma was separated by centrifugation (10 min at 2000 rpm) and kept at −30 °C until being assayed for cytokines (Figure 1B) [21,22].

### 2.5. Test Methods-ELISA and Electrochemiluminescence Assay

A Thromboxane B2 Parameter Assay Kit (Catalog no. KGE011:) and Prostaglandin E2 Parameter Assay Kit (Catalog no. KGE004B:) from R&D Systems, Inc. a Bio-Techne Brand were analyzed by ELISA using serum and plasma, respectively. The ELISA was performed according to the manufacturer’s guidelines, including the relevant controls. The plates were read using a microplate ELISA reader (VersaMax, Molecular Devices, USA). Changes in the inflammatory plasma cytokines (IL-1α, IL-1β, IL-6, IL-16, TNFα) were analyzed in duplicate using the V-Plex Plus human cytokine kits (Mesoscale, Rockville, MA, USA, Catalog no. K15249D) in accordance with the manufacturer’s guidelines. The plates were read using the MSD QuickPlex (SQ120) Plate Reader (Mesoscale Discovery), and the data were analyzed using the MSD Discovery Workbench software, as detailed in [23].

### 2.6. Statistical Analysis

With an explorative approach and no indicators of the expected size of the endpoints, no power calculations were performed prior to the study launch. Further, due to the explorative approach of the study and the limited sample size, non-parametric tests were performed. Wilcoxon signed-rank tests were performed to compare the concentrations of IL-1α, IL-1b, IL-6, IL-16, TNFα, TXB2, and PGE2 at baseline, with their concentrations, respectively, one hour after carrot juice intake. This was repeated for the samples stimulated with LPS or ASA. The significance level was set at 5%. Box plots were created to illustrate the distribution of the measurements. All of the data management and statistical analyses were performed in the SAS software version 9.4 (SAS Institute Inc. SAS 9.4. Cary, NC, USA) and R statistical software package version 3.6.1 (R Core Team, Vienna, Austria).

## 3. Results

Figure 1 shows the study design to illuminate the flow of the study from the blood sampling to the analysis of the blood samples *ex vivo*.

### 3.1. Levels of TXB2 in Coagulated Blood before and after Intake of Carrot Juice

**Figure 2:**
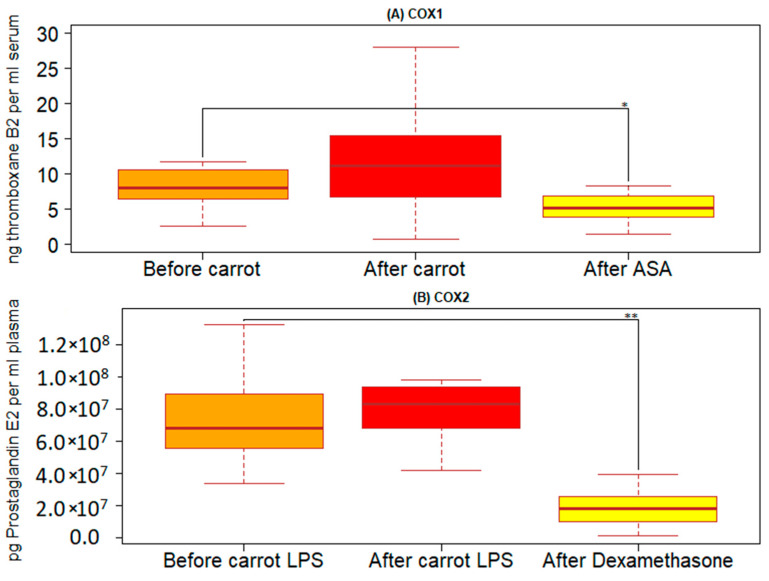
(**A**) TXB2 serum level in blood samples after the clotting process. Orange box blood samples taken before intake of carrot juice. Red box blood samples taken after intake of carrot juice. Yellow box blood samples taken before carrot intake adding ASA before the clotting process for 1h at RT. (**B**): PGE2 plasma level in blood samples after LPS stimulation. Orange box blood samples taken before intake of carrot juice adding LPS before incubation for 24 h at 37 °C. Red box blood samples taken after intake of carrot juice adding LPS before incubation for 24 h at 37 °C. Yellow box blood samples taken before carrot intake adding LPS and Dexamethasone before incubation for 24 h at 37 °C. Sample size n = 14. Cyclooxygenase (COX), Tromboxane (TX), Prostaglandin (PL), Acetyl Salicyl Acid (ASA), Lipopolysacharide (LPS). *, *p* below 0.05; **, *p* below 0.01.

The human serum samples showed no significant changes in their TXB2 levels 1 h after carrot juice intake compared to the baseline levels (before carrot juice intake) (Figure 2A). 

If ASA was added to the blood samples (before carrot juice intake) before the clotting process, we observed a statistically significant decrease in serum TXB2 (*p* < 0.02). 

### 3.2. Levels of PGE2 and Inflammatory Markers in Plasma before and after Intake of Carrot Juice

The analysis of the plasma samples before and after the intake of carrot juice, without LPS stimulation, showed no statistically significant effect on PGE2, IL-1α, IL-1β, IL-6, IL-16, and TNFα (Table 1). 

If LPS was added to the blood samples taken before and after carrot juice intake, the LPS induced an increased level of PGE2 in the blood. Analyzing the data from before and after carrot juice intake resulted in no statistically significant effect on PGE2 in this *ex vivo* setup. This is illustrated by a box plot in Figure 2B and Table 1. If Dexamethasone was added to the blood samples (before carrot juice intake) together with LPS, we observed a significant decrease in plasma PGE2 (*p* = 0.0081).

**Figure 3:**
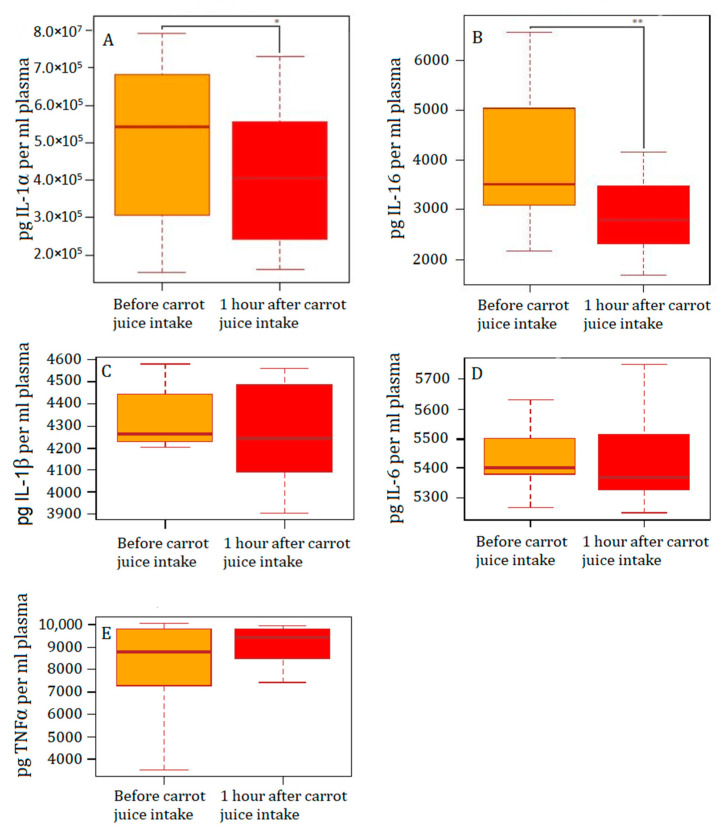
Plasma levels of cytokines after stimulation with LPS for 24h at 37 C. Yellow boxes LPS stimulated blood before intake of carrot juice. Red boxes LPS stimulated blood after intake of carrot juice. (**A**): IL-1α, (**B**): IL-16, (**C**): IL-1β, (**D**): IL-6, (**E**): TNFα. Sample size n = 14. Interleukin (IL), Tumor Necro-sis Factor (TNF), Lipopolysachride (LPS).*, *p* below 0.05; **, *p* below 0.01.

The comparisons of the concentrations before and after the carrot juice intake revealed significant inhibitions of IL-1α (Figure 3A) and IL-16 (Figure 3B) in the setup of this study (Table 1). No significant inhibitions of IL-1β (Figure 3C), IL-6 (Figure 3D), or TNFα (Figure 3E) were evident in the *ex vivo* setup (Table 1). The complete data set is included in Supplementary File S1. 

## 4. Discussion

For the past decade, *in vitro* experiments and animal studies have indicated that the FaOH and FaDOH from carrots are potent inhibitors of inflammation and cancer development in the colorectal intestine. Previously, in long-term studies, the daily intake of FaOH/FaDOH from carrots showed a significant prevention of neoplasm transformation in the colorectal intestine in rats primed by the carcinogen Azoxymethane. The results showed significant downregulation in COX-2, IL-6, NFκβ, and TNFα gene expression (14). However, it was not possible to translate these animal studies in our study conducted in healthy human individuals, as the induction of inflammation *ex vivo* did not downregulate the protein levels of COX-2, TNF and IL-6; instead, it downregulated IL-1α and IL-16. There are several differences in the setup of preclinical animal studies compared to the present human *ex vivo* study. The translation from rat studies to human studies is the most obvious reason for the different results. Furthermore, the long-time rat studies were conducted over 26 weeks, with an intake of a small amount of FaOH and FaDOH throughout the day [15]. In the *ex vivo* study, the carrot intake was administrated orally to human volunteers within 5 min, resulting in a peak concentration of FaOH after 1 h [5]. This indicates that the mode of action is different in the everyday intake of carrots over a long time compared to one large quantum intake of carrots. 

The increased production of TXB2 reveals increased COX1 activity in the samples collected 1h after the carrot juice intake compared to the baseline levels, at a timepoint when the peak concentration of FaOH has previously been shown [5]. The activity of COX-1 was significantly inhibited by adding ASA to the blood samples *ex vivo* before the coagulation process. 

IL-1α is constitutively present as a precursor in all healthy tissues [24], increasing markedly upon exposure to pathogens and neoplastic transformation [18]. Accumulating evidence suggests that IL-1α is involved in cancer pathogenesis [25] and highly expressed by the epithelium of the entire gastrointestinal tract [24]. Due to its ubiquitous intracellular expression, potent activity, and bioavailability, IL-1α has emerged as a major drug target in inflammatory diseases and cancer. In the present study, the intake of carrot juice rich in FaOH and FaDOH significantly inhibits the secretion of IL-1α from LPS-activated leukocytes (Figure 3A, and Table 1). This effect was not seen in the blood samples not subjected to LPS stimulation.

In addition, IL-16 a multifunctional cytokine that plays a key role in inflammatory diseases, as well as in the development and progression of various cancers, e.g., breast and gastrointestinal cancers [26]. IL-16 may play a major role in patients with colorectal or gastric cancer, and the plasma concentration has been shown to increase with cancer progression [25,27]. In our study, the oral carrot juice intake significantly decreased the secretion of IL-16 after *ex vivo* stimulation with LPS (Figure 3B, and Table 1). This effect was not seen in the blood samples not stimulated with LPS.

For the past decade, *in vitro* experiments and animal studies have indicated that FaOH and FaDOH from carrots are inhibitors of inflammation and, thereby, most likely inhibit cancer development in the colorectal intestine. The main focus has been on COX-2, as well as IL-1β, IL-6, and TNFα, as these inflammatory markers have been shown to be downregulated in gene expression studies. However, a significant inhibition was only observed of IL-1α and IL-16 on the protein level in our *ex vivo* setup on humans. Both IL-1α and IL-16 are key players of inflammation and colorectal cancer development. Therefore, there is a need for clinical studies with further investigation of these *ex vivo* results. Specifically, studies with several samples taken from each subject, in order to replicate the results, are needed.

## 5. Conclusions

In the present study, we have shown that the intake of carrot juice decreased the secretion of a central cytokine in inflammation related to cancer development: IL-1α and IL-16 in blood samples stimulated with LPS *ex vivo*, although only one sample was obtained from each subject. No inhibition of COX-2, as well as IL-1β, IL-6, and TNFα, was observed in this setup. 

## Figures and Tables

**Table 1:** Overview of *ex vivo* results. Interleukin (IL), Tumor Necrosis Factor (TNF), Prostaglandin (PL), Lipopolysacharide (LPS).

	Ex Vivo Normal Conditions			Ex Vivo Inflammation (LPS)		
Biomarker	No Carrots Median	Carrots Median	*p*-Value	No Carrots Median	Carrots Median	*p*-Value
IL-1α (pg/mL)	1441	1496	*p* = 0.6812	542,144	405,623	*p* = 0.0419
IL-1β (pg/mL)	70	120	*p* = 0.9032	4260	4268	*p* = 0.9032
IL-6 (pg/mL)	358	947	*p* = 04263	5431	5422	*p* = 0.9515
IL-16 (pg/mL)	2042	2126	*p* = 0.9032	3520	2794	*p* = 0.0085
TNFα(pg/mL)	18	18	*p* = 0.5830	8099	8479	*p* = 0.1531
PgE2 (pg/mL)	1,607,925	1,668,736	*p* = 0.1294	68,154,921	83,403,951	*p* = 0.1040

## Data Availability

Not applicable.

## Supplementary Materials

The following supporting information can be downloaded at: https://www.mdpi.com/article/10.3390/nu15030632/s1. Table S1: COX-1; Table S2: COX-2; Table S3: IL-1α; Table S4: IL-1β; Table S5: IL-6; Table S6: IL-16; Table S7: TNFα.
