# Supplementary material for: Effect of Oral Intake of Carrot Juice on Cyclooxygenases and Cytokines in Healthy Human Blood Stimulated by Lipopolysaccharide
Source: Nutrients. 2023 Jan 26;15(3):632. doi: 10.3390/nu15030632 (PMC9920447; doi:10.3390/nu15030632)
Supplement: Supplementary file 1 [file nutrients-15-00632-s001.zip › nutrients-2139775-supplementary.pdf]

# Supplementary file S1

**Table S1:** COX-1

| Participant | Gender | No carrots ng/ml | Carrots ng/ml | No carrots ASA ng/ml |
|---|---|---|---|---|
| 1 | M | 6,87 | 11,25 | 4,31 |
| 2 | F | 10,63 | 10,36 | 4,32 |
| 3 | F | 8,92 | 16,08 | 5,93 |
| 4 | F | 11,77 | 28,00 | 13,89 |
| 5 | F | 5,01 | 11,08 | 3,94 |
| 6 | F | 6,02 | 6,78 | 6,75 |
| 7 | M | 11,35 | 15,41 | 2,94 |
| 8 | M | 8,05 | 4,20 | 6,51 |
| 9 | F | 29,63 | 25,11 | 3,94 |
| 10 | F | 6,48 | 8,70 | 8,31 |
| 11 | F | 2,55 | 0,78 | 1,40 |
| 12 | F | 7,94 | 15,29 | 8,67 |
| 13 | M | 7,19 | 5,07 | 3,88 |
| 14 | M | 10,16 | 11,96 | 6,98 |

**Table S2:** COX-2

| Participant | Gender | No carrots pg/ml | Carrots pg/ml | No carrots LPS added pg/ml | Carrots LPS added pg/ml | No carrots LPS and Dexa added pg/ml |
|---|---|---|---|---|---|---|
| 1 | M | 466739 | 70542408 | 3369823 | 86101725 | 20508724 |
| 2 | F | 1982854 | 77767535 | | 90536988 | |
| 3 | F | 1440879 | 60315102 | 1321057 | 57246236 | 10154718 |
| 4 | F | 1096211 | 33696773 | 1895064 | 42010169 | 1782396 |
| 5 | F | 880892 | 41306814 | 1027119 | 49727875 | 1097463 |
| 6 | F | 1701529 | 48103345 | 1394487 | 68351010 | 1226752 |
| 7 | M | 7347504 | 65665182 | 14423545 | 98618868 | 19782173 |
| 8 | M | 1788805 | 82579419 | 1442408 | 82725732 | 25541120 |
| 9 | F | 2508834 | 89849674 | 7703048 | 84082169 | 18143085 |
| 10 | F | 1944856 | 65767433 | 2112409 | 70168439 | 14665148 |
| 11 | F | 332885 | 55876449 | 892063 | 98246231 | 1,13E+08 |
| 12 | F | 1514320 | 92878821 | 966018 | 82670323 | 10123442 |
| 13 | M | | 1,1E+08 | 405094 | 1,36E+08 | 39698906 |
| 14 | M | 1783695 | 1,33E+08 | 1955666 | 93746813 | 55485768 |

**Table S3:** IL-1α

| Participant | Gender | No carrots<br><br>pg/ml | Carrots<br><br>pg/ml | No carrots<br>LPS<br>pg/ml | Carrots<br>LPS<br>pg/ml |
|---|---|---|---|---|---|
| 1 | M | 1334 | 3178 | 792098 | 451495 |
| 2 | F | 17197 | 1054 | 594967 | 359751 |
| 3 | F | 816 | 788 | 304987 | 240814 |
| 4 | F | 1167 | 2606 | 248684 | 268910 |
| 5 | F | 1090 | 1036 | 190167 | 162474 |
| 6 | F | 979 | 973 | 592583 | 359557 |
| 7 | M | 5501 | 19483 | 491705 | 499208 |
| 8 | M | 5773 | 4575 | 599627 | 729206 |
| 9 | F | 1953 | 5089 | 471372 | 461277 |
| 10 | F | 1435 | 1489 | 345003 | 205001 |
| 11 | F | 1397 | 848 | 731978 | 558499 |
| 12 | F | 1977 | 2063 | 720162 | 726826 |
| 13 | M | 1447 | 1157 | 152859 | 161732 |
| 14 | M | 1451 | 1504 | 682974 | 556847 |

**Table S4:** IL-1β

| Participant | Gender | No carrots<br><br>pg/ml | Carrots<br><br>pg/ml | No carrots<br>LPS<br>pg/ml | Carrots<br>LPS<br>pg/ml |
|---|---|---|---|---|---|
| 1 | M | 12,20 | 327,10 | 4264,95 | 4228,18 |
| 2 | F | 262,78 | 4,70 | 4238,80 | 4259,22 |
| 3 | F | 4,06 | 3,34 | 4447,75 | 4429,07 |
| 4 | F | 44,80 | 156,48 | 4262,55 | 4206,87 |
| 5 | F | 2,65 | 2,18 | 3826,33 | 4038,47 |
| 6 | F | 4,89 | 4,04 | 4203,10 | 4316,36 |
| 7 | M | 268,24 | 790,10 | 4341,85 | 4515,94 |
| 8 | M | 187,05 | 116,06 | 4452,19 | 4561,16 |
| 9 | F | 86,35 | 198,86 | 4262,51 | 4143,33 |
| 10 | F | 9,93 | 1,75 | 3718,16 | 3903,19 |
| 11 | F | 27,46 | 3,99 | 4500,38 | 4509,34 |
| 12 | F | 62,98 | 60,10 | 4583,52 | 4484,52 |
| 13 | M | 9,19 | 14,74 | 4226,84 | 4093,52 |
| 14 | M | 2,70 | 2,85 | 4305,19 | 4065,30 |

**Table S5:** IL-6

| Participant | Gender | No carrots<br>pg/ml | Carrots<br>pg/ml | No carrots<br>LPS<br>pg/ml | Carrots<br>LPS<br>pg/ml |
|---|---|---|---|---|---|
| 1 | M | 17,53 | 2038,28 | 5379,97 | 5513,18 |
| 2 | F | 959,10 | 3,60 | 5613,30 | 5349,61 |
| 3 | F | 4,10 | 4,46 | 5354,41 | 5246,67 |
| 4 | F | 177,98 | 1554,41 | 5497,29 | 5609,63 |
| 5 | F | 3,24 | 3,02 | 5267,95 | 5313,53 |
| 6 | F | 3,97 | 3,41 | 5384,83 | 5360,01 |
| 7 | M | 1423,65 | 5105,96 | 5629,21 | 5750,33 |
| 8 | M | 1801,47 | 1172,55 | 5503,03 | 5426,75 |
| 9 | F | 181,46 | 2829,90 | 5401,25 | 5621,81 |
| 10 | F | 6,05 | 3,39 | 5413,21 | 5375,34 |
| 11 | F | 24,04 | 4,03 | 5374,92 | 5338,72 |
| 12 | F | 403,69 | 530,62 | 5431,14 | 5400,43 |
| 13 | M | 4,08 | 6,62 | 5402,93 | 5325,00 |
| 14 | M | 2,86 | 2,84 | 5383,43 | 5276,17 |

**Table S6:** IL-16

| Participant | Gender | No carrots<br>pg/ml | Carrots<br>pg/ml | No carrots<br>LPS<br>pg/ml | Carrots<br>LPS<br>pg/ml |
|---|---|---|---|---|---|
| 1 | M | 3954,10 | 1567,58 | 6547,58 | 2315,57 |
| 2 | F | 1726,61 | 3034,17 | 2417,43 | 3154,39 |
| 3 | F | 1736,13 | 1127,05 | 3564,32 | 2325,84 |
| 4 | F | 1874,61 | 1734,83 | 3474,83 | 2775,61 |
| 5 | F | 2227,17 | 2527,02 | 3097,91 | 2200,55 |
| 6 | F | 2217,12 | 2213,52 | 5027,25 | 3042,91 |
| 7 | M | 2008,05 | 3178,59 | 3081,54 | 3908,23 |
| 8 | M | 1150,58 | 1723,19 | 2351,30 | 2742,16 |
| 9 | F | 1710,07 | 1512,88 | 2183,83 | 2417,73 |
| 10 | F | 2396,53 | 3727,30 | 4629,07 | 3476,67 |
| 11 | F | 1445,54 | 934,02 | 3275,46 | 1688,95 |
| 12 | F | 2075,51 | 2038,74 | 5235,07 | 3698,06 |
| 13 | M | 3438,92 | 3493,18 | 3867,95 | 2812,26 |
| 14 | M | 4785,63 | 2485,56 | 6007,96 | 4155,71 |

**Table S7:** TNFα

| Participant | Gender | No carrots<br><br>pg/ml | Carrots<br><br>pg/ml | No carrots<br>LPS<br>pg/ml | Carrots<br>LPS<br>pg/ml |
|---|---|---|---|---|---|
| 1 | M | 9,76 | 38,51 | 7289,24 | 9399,40 |
| 2 | F | 37,01 | 4,75 | 8087,47 | 9052,15 |
| 3 | F | 3,95 | 3,55 | 8638,40 | 9264,21 |
| 4 | F | 41,94 | 41,28 | 5468,03 | 8485,35 |
| 5 | F | 4,12 | 5,03 | 3505,99 | 3628,27 |
| 6 | F | 7,12 | 4,92 | 9750,26 | 9565,62 |
| 7 | M | 60,02 | 63,17 | 8917,50 | 9483,01 |
| 8 | M | 25,75 | 17,86 | 9053,54 | 9408,05 |
| 9 | F | 18,10 | 42,27 | 7498,38 | 7416,39 |
| 10 | F | 4,92 | 3,47 | 5591,09 | 3523,72 |
| 11 | F | 17,69 | 4,67 | 9845,79 | 9844,30 |
| 12 | F | 12,52 | 12,19 | 10061,35 | 9782,18 |
| 13 | M | 5,24 | 5,30 | 9856,51 | 9955,74 |
| 14 | M | 2,95 | 2,96 | 9826,49 | 9894,36 |
